# Efficient detection of deformation-induced microstructural modifications in polycrystalline micropillars using scanning X-ray nanodiffraction

**DOI:** 10.1107/S160057672500946X

**Published:** 2026-02-01

**Authors:** Anton Davydok, Kritika Singh, Surya Snata Rout, Christina Krywka

**Affiliations:** a Institute of Materials Physics Helmholtz-Zentrum Hereon GmbH Outstation at DESY, Notkestr. 85 Hamburg22607 Germany; b School of Earth and Planetary Sciences National Institute of Science Education and Research HBNI Jatani Khordha Odisha752050 India; c Homi Bhabha National Institute Training School Complex Anushaktinagar Mumbai400094 India; Institut de Recherche sur les Céramiques, France

**Keywords:** polycrystalline micropillars, X-ray nanodiffraction, X-ray fluorescence, nanomechanical testing, X-ray data analysis, deformation, microstructure

## Abstract

Microstructural characterization of polycrystalline micropillars remains a significant challenge, particularly under time constraints such as those encountered during *in situ* or other time-sensitive experimental conditions, where appropriate data checks might assist in taking the right decision and have influence on the outcome of the experiment. In this study, we present a fast and efficient method for estimating local structural properties using scanning X-ray nanodiffraction—a technique widely employed in various dynamic and static micro- and nanoscale material investigations. The analysis targets the strongest diffraction peaks within the scattering pattern to extract essential information on grain orientation, size and lattice strain, while excluding weaker signals to streamline processing. As a case study, a γ-TiAl-based micropillar (Ti–46.5Al–5Nb), fabricated via Xe^+^ plasma focused-ion-beam milling, was analyzed before and after 10% uniaxial compression. The micropillar’s grain size significantly exceeded the X-ray beam size (∼300 nm^2^), and its known crystallographic orientation enabled accurate tracking of structural evolution. A direct point-to-point comparison between the undeformed and compressed states revealed localized microstructural changes associated with plastic deformation. This approach provides a rapid and reliable means of assessing microstructural evolution and demonstrates high potential for *in situ* and *operando* investigations of small-scale materials.

## Abstract 2

A rapid scanning X-ray nanodiffraction method is demonstrated to efficiently assess local microstructural changes in polycrystalline micropillars during deformation, enabling real-time insights.

## 1. Introduction

Most of the metallic structural materials that are currently used in civil engineering, aerospace applications, electronics and various other industries are polycrystalline (Kear & Thompson, 1980 ▸; Vollmer *et al.*, 2021 ▸; Yazyev & Louie, 2010 ▸). Large-scale components are composed of materials with diverse grain sizes and dimensions. Moreover, specific processing techniques, such as annealing, can significantly alter their properties (Amram & Schuh, 2018 ▸). In many metallic alloys, local composition, grain size and distribution can be precisely controlled, directly influencing the final product’s characteristics and, consequently, its industrial applications (Lu *et al.*, 2020 ▸). A detailed investigation of small-scale effects and microstructural features in polycrystalline materials is crucial for their successful implementation and adaptation to industrial demands. Such studies require advanced characterization techniques to ensure reliable assessment before large-scale application.

Microscopy is the primary method for analyzing small-scale structures and effects. Modern optical and electron microscopy provide high-resolution imaging, detailed surface analysis and material-composition characterization (Tian *et al.*, 2025 ▸). However, both techniques are inherently surface sensitive and do not provide access to internal structures. This limitation can be partially addressed using electron backscatter diffraction (EBSD) (Wang *et al.*, 2023 ▸), but the method requires sample preparation that may alter the original properties and the process itself is destructive. Transmission electron microscopy (Meng *et al.*, 2024 ▸) is another technique for internal structural analysis, but it requires complex sample preparation, is restricted to a small volume of the sample and operates in a high-vacuum environment.

An alternative approach to traditional microscopy-based characterization techniques is X-ray diffraction (XRD) using synchrotron radiation, which has emerged as a powerful tool for analyzing polycrystalline materials. It offers high spatial resolution, non-destructive probing, and the unique ability to access both surface and internal structural characteristics. Recent advances in X-ray optics have enabled beam focusing down to the micrometre (Guilherme *et al.*, 2012 ▸) and nanometre scale (Singhapong *et al.*, 2024 ▸), allowing researchers to overcome many of the spatial limitations inherent in conventional methods.

One of the principal strengths of synchrotron-based XRD lies in its capacity for detailed non-destructive analysis of complex multi-phase and multi-scale materials. For example, Bonnin *et al.* (2014 ▸) employed high-resolution XRD computed tomography combined with an X-ray nanoprobe to investigate a 20 µm solidified atomized γU-Mo particle. This multimodal technique enabled the identification of a minority U(C,O) phase with sub-micrometre grain size and revealed its precipitation mechanism. However, achieving such high-quality results required the integration of multiple advanced tech­niques and significant computational resources, underscoring a major limitation of these methods: the complexity of data acquisition and analysis.

A similar challenge was encountered by Henningsson *et al.* (2024 ▸), who applied advanced X-ray imaging to map the three-dimensional structure of an aluminium polycrystal deformed to its ultimate tensile strength (32% elongation). This enabled precise evaluation of inter- and intragrain stress distributions, which is essential for understanding mechanical performance under extreme conditions. However, the success of this approach depended heavily on sophisticated imaging algorithms and high-resolution optics, making it both technically demanding and resource intensive.

Another noteworthy application of synchrotron-based multimodal X-ray techniques is in the field of photovoltaics, as shown by Ulvestad *et al.* (2019 ▸). These researchers utilized focused nanobeam X-ray microscopy to concurrently map chemical composition, lattice structure and charge-collection efficiency in a Cu(In,Ga)Se_2_ solar cell. This comprehensive analysis provided critical insights into how grain boundaries impact device performance, informing strategies for solar-cell optimization. Nonetheless, the integration of multiple X-ray modalities introduced challenges related to instrument calibration, data alignment and overall experimental complexity.

A more recent development is nano three-dimensional X-ray diffraction microscopy (nano-3DXRD), as demonstrated by Oh *et al.* (2025 ▸). This technique employs a nanofocused synchrotron beam to scan and reconstruct grain structures and internal strain fields within polycrystalline materials. It enables fully three-dimensional non-destructive imaging of individual grains at sub-micrometre resolution, providing rich detail on local deformation behavior and grain-boundary mechanics. By resolving both intragranular and intergranular features, nano-3DXRD marks a significant advancement in the understanding of nanoscale mechanical processes. However, its application is still constrained by lengthy data-acquisition times, high computational demands for tomographic reconstruction and the necessity for exceptional beamline stability—factors that currently limit its accessibility to well-equipped synchrotron facilities and carefully prepared specimens.

Laue microdiffraction is a powerful technique for orientation and strain mapping, particularly in *in situ* applications. However, its use in polycrystalline or highly strained materials can be limited by challenges such as intensity normalization in polychromatic beams, detector saturation and complex spot shapes that complicate indexing. These limitations are especially pronounced when grain sizes approach or fall below the beam size, leading to overlapping reflections and reduced spatial resolution (Purushottam Raj Purohit *et al.*, 2024 ▸; Gürsoy *et al.*, 2022 ▸).

While advanced XRD-based techniques can provide highly detailed non-destructive 3D grain reconstructions of polycrystalline materials, they are often time consuming and require significant experimental and computational resources. In contrast, the method presented in this study offers a less comprehensive but substantially more accessible alternative. Using scanning X-ray nanodiffraction, we target the strongest diffraction peaks within the scattering pattern to extract essential information on grain orientation, size and lattice strain, while excluding weaker signals to streamline processing. This approach enables fast and straightforward estimation of local structural prop1erties under specific conditions, when the material has a known crystallographic orientation and grain sizes larger than the X-ray beam size. These constraints allow individual strong diffraction reflections to be attributed to well-defined illuminated volumes within the sample. Although this approach cannot rival full 3D characterization in detail, it requires significantly fewer resources and is well suited for rapid structural assessment. We demonstrate its utility by analyzing scattering patterns from γ-TiAl micropillars before and after uniaxial compression, enabling detection of deformation-induced microstructural changes. The potential impact of ion implantation from focused-ion-beam (FIB) milling and its interaction with mechanical loading is also addressed.

## 2. Experimental setup

### 2.1. Sample preparation

The material used in this study is an intermetallic Nb-containing titanium aluminide (Ti–46.5Al–5Nb) that is extensively used in lightweight, high-strength and high-temperature applications (*e.g.* turbine blades in an aircraft engine). It has been widely studied at Helmholtz-Zentrum Hereon (Fröbel & Laipple, 2020 ▸). The alloy consists of two intermetallic phases: γ(TiAl) and α_2_(Ti_3_Al). The micropillar was prepared using a TESCAN AmberX Plasma (Xe^+^) focused-ion-beam scanning electron microscope (PFIB-SEM) with 30 keV ion energy and 10 nA ion-beam current while maintaining a 90° incident angle.

### 2.2. Synchrotron measurements

**Figure 1:**
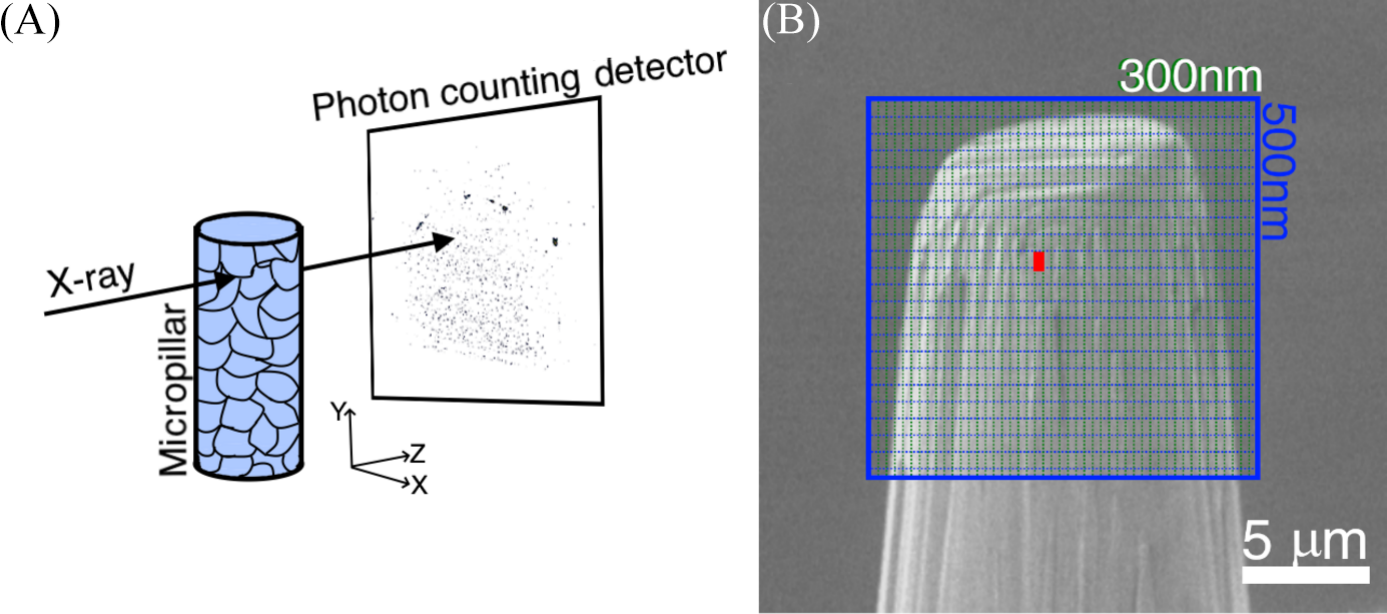
(*A*) Schematic image of the experimental setup. (*B*) SEM image of the top part of the micropillar with marked scanned area and corresponding step size during scans.

Synchrotron measurements were conducted in two sessions at the Nanofocus Endstation of the P03 beamline at PETRA III, DESY (Hamburg, Germany) (Krywka *et al.*, 2013 ▸). The first session focused on assessing the effects of FIB milling protocols on the local crystal structure of metallic micropillars, as detailed in our previous study (Singh *et al.*, 2023 ▸). The second session involved repeating the same measurements after uniaxial compression of the micropillar. In both sessions, the synchrotron beam was set to a photon energy of 12.95 keV and focused to a 300 × 300 nm spot using Kirkpatrick–Baez mirrors. Diffraction patterns were recorded using a two-dimensional Eiger 9M detector with a pixel size of 75 × 75 µm, positioned ∼18 cm downstream of the sample. Simultaneously, an Amptek SSD 123 detector located 3 cm downstream collected X-ray fluorescence signals. Two-dimensional raster scans were performed on the micropillar in both undeformed and compressed states, using a step size of 300 × 500 nm (horizontal × vertical). No sample rotation was applied during the measurements; instead, only 2D spatial maps were recorded. These maps, capturing the spatial distribution of the strongest diffraction signals, are further evaluated and analyzed in this article. Fig. 1 ▸(*A*) illustrates the experimental geometry and Fig. 1 ▸(*B*) shows the scan area, where the defined grid was used to systematically collect scattering signals across the micropillar surface. Obtained scanning X-ray nanodiffraction (SXND) and X-ray fluorescence data were analyzed and visualized by using the *pyFAI* library (Kieffer & Karkoulis, 2013 ▸) and custom MATLAB scripts. The diffraction data were analyzed in terms of the scattering-vector magnitude *q*, covering a range from 0 to 40 nm^−1^, with a photon energy of 12.95 keV and the above-mentioned sample-to-detector distance. Due to shadowing from the sample holder, the azimuthal angle φ was limited to 0–180°; nevertheless, strong signal contribution was obtained at every micropillar scan spot. Scanning the area shown in Fig. 1 ▸(*B*), with an exposure time of 5 s per point for a total of 1675 points, including machine operation and detector readouts, required ∼3 h. An additional hour was needed for data processing, including scattering-pattern integration, peak identification and fitting, and constructing the final 2D parameter distribution map, assuming that all preparatory steps such as experimental-setup calibration and integration area definition had already been completed. While the full scanned area spans ∼10 × 10 µm [Fig. 1 ▸(*B*)], the analysis here focuses on the upper 3 µm region, where Xe^+^ ion implantation and deformation effects are most pronounced. The complete dataset, including full vertical coverage, is provided in Fig. S1 of the supporting information.

### 2.3. Mechanical testing

The micromechanical tests were performed *in situ* in the PFIB-SEM at an acceleration voltage of 2 keV. The microcompression of the TiAl micropillar was conducted using a FemtoTools FT-NMT04 nanoindenter with a 50 µm diameter diamond flat punch. The nanoindenter was operated in displacement control mode at a constant strain rate of 0.01 s^−1^, aiming for compression down to 10% of the micropillar height. The alignment of the micropillar was performed by using different sample projections and an iterative search of the contact surface between the tip and the sample bulk part next to the micropillar.

### 2.4. EBSD analysis

The EBSD characterization was performed on the top part of the middle slice of the micropillar at 70° tilt in a Nova NanoSEM 450, FEI Thermofisher, with an electron energy of 20 keV using a C-Nano EBSD CMOS detector, Oxford Instruments (Stierle *et al.*, 2016 ▸). For the orientation and phase analysis, the micropillar was sliced using the PFIB-SEM parallel to the length of the micropillar until half of the micropillar was removed.

## 3. Results

**Figure 2:**
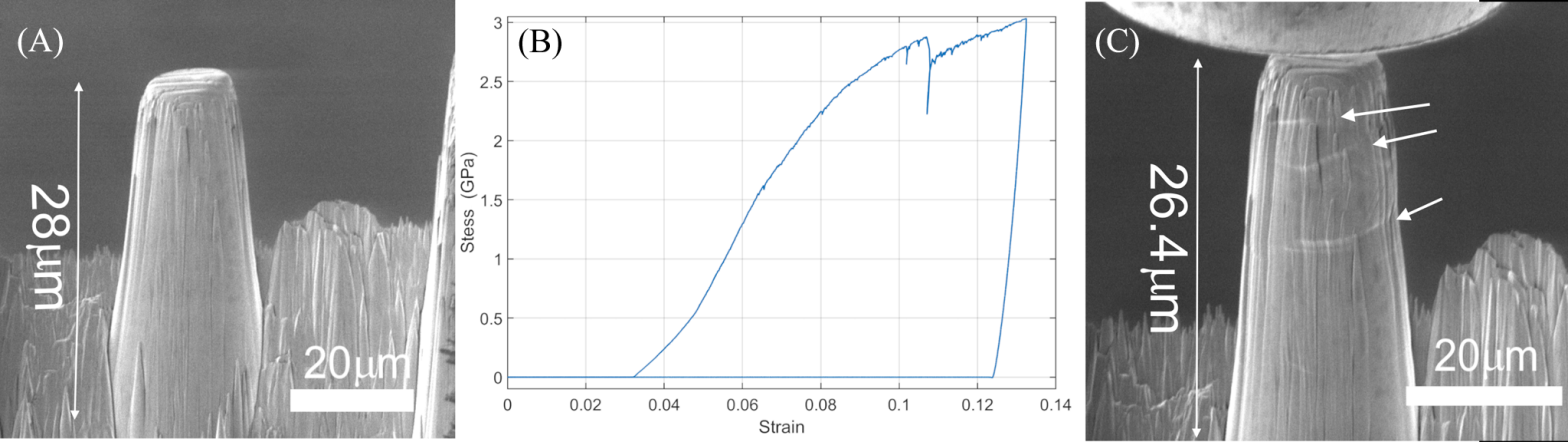
SEM images (*A*) before and (*C*) after deformation. (*B*) Strain–stress curve of compression test on the micropillar.

Fig. 2 ▸(*A*) presents a secondary electron (SE) image of the pristine micropillar used in this study. The micropillar has an initial height of 28 µm and a top diameter of 10 µm, resulting in an aspect ratio of 3, which is typical for nanomechanical testing (Uchic *et al.*, 2004 ▸). The structure appears solid and homogeneous, with no visible cracks or defects on the surface, except for minor imperfections due to FIB milling. These imperfections are attributed to the coarse high-beam-current milling protocol used for Xe^+^ implantation investigation (Singh *et al.*, 2023 ▸). Fig. 2 ▸(*B*) displays the stress–strain (σ–ɛ) curves obtained from uniaxial compression tests on the micropillar. The maximum stress reached 3 GPa at a strain of 10%, corresponding to a displacement of 3 µm. During loading, plastic deformation occurred, accompanied by two distinct pop-in events: a minor one at a strain of 0.103 and a more pronounced one at 0.108. The initial linear portion of the stress–strain curve up to a strain of ∼0.8% corresponds to the elastic regime. Although the displacement axis includes the instrument-compliance contribution, the apparent stiffness is consistent with Young’s modulus in the range of 350–400 GPa, which agrees well with literature values for γ-TiAl alloys (340–420 GPa) (Fröbel & Laipple, 2020 ▸). The deviation from linearity marks the onset of plasticity, and the yield strength can be estimated at ∼2.6 GPa. Beyond this point, the curve exhibits limited strain hardening, reaching a maximum compressive stress of ∼3 GPa at 10% strain. Such behavior is typical for intermetallic TiAl-based micropillars. The two pop-in events observed at strains of 0.103 and 0.108 probably correspond to sudden dislocation bursts or localized slip activation, reflecting the transition from homogeneous to localized deformation. Following these events, complete unloading was observed. A detailed analysis of the nanomechanical data from this compression test will be published separately. Fig. 2 ▸(*C*) presents an SE image of the micropillar immediately after flat-punch extraction, with the punch still partially visible at the top of the image. The micropillar remains upright but exhibits a reduced height of 26.4 µm, along with horizontal cracks on its facets (indicated by arrows), which were not observed in the pristine state. To gain further structural insights, X-ray nanodiffraction measurements were performed before and after deformation.

**Figure 3:**
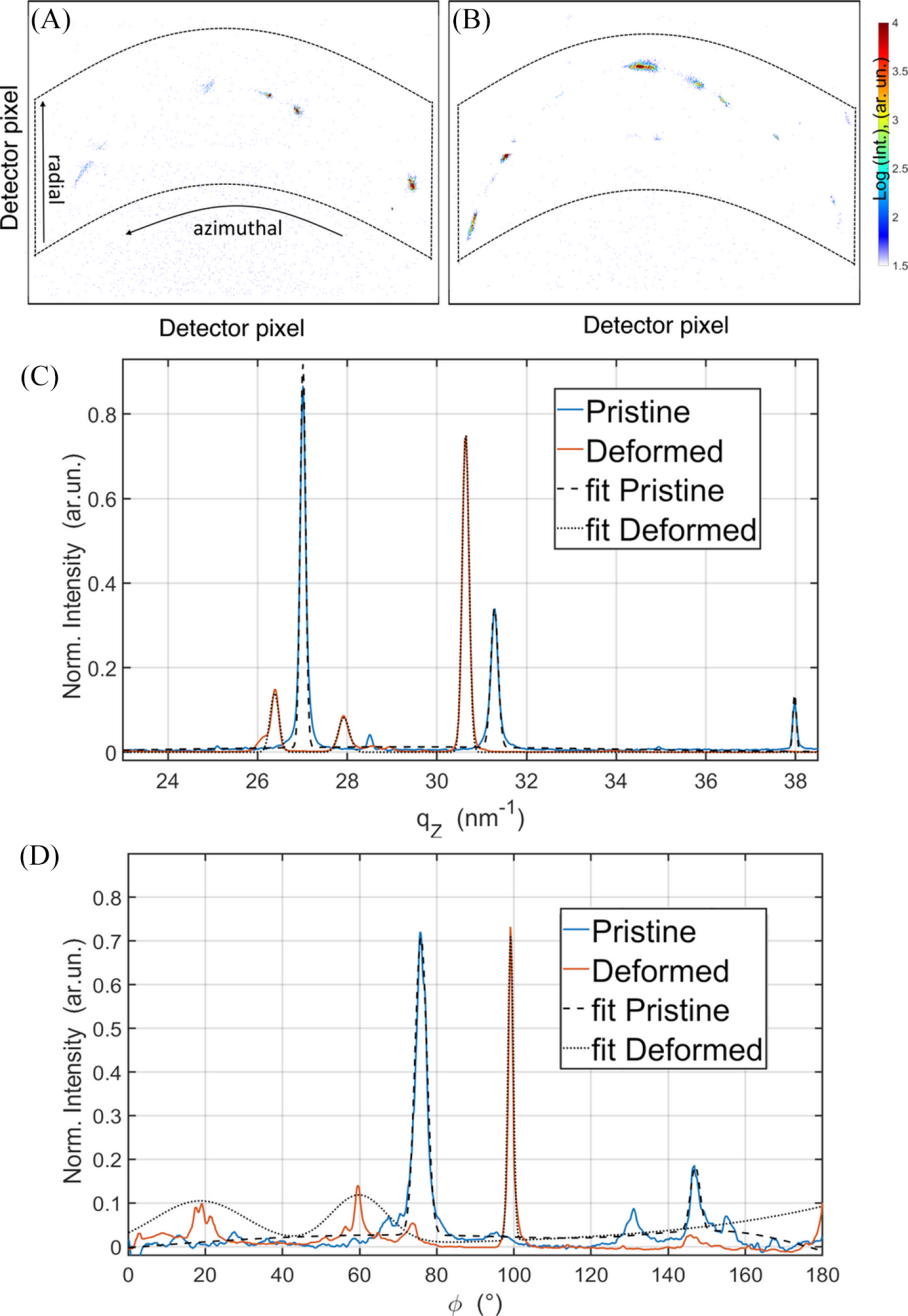
Scattering patterns recorded inside the micropillar at the same spot in (*A*) pristine state and (*B*) after deformation, (*C*) azimuthally integrated intensity curves of the above-shown scattering patterns, and (*D*) radially integrated intensity curves.

Structural changes in the micropillars were analyzed using SXND. Both the pristine and post-mortem measurements were carried out on the same micropillar, which remained tightly mounted in a SEM-stub-based holder throughout the experiments. The identical measurement position was verified using SEM and optical images, as well as fiducial references from the pillar borders and Xe ion implantation pattern, ensuring consistent orientation and spatial alignment between datasets. Typical X-ray scattering patterns obtained during these measurements are shown in Figs. 3 ▸(*A*) and 3 ▸(*B*). These patterns correspond to a polycrystalline material with multiple phases and orientations of crystallites, indicated by the presence of several spotted diffraction rings. In this study, a nanofocused X-ray beam with a size of 300 × 300 nm, which is considerably smaller than the lateral grain dimensions of the micropillars (ranging from 3 to 5 µm) (Fröbel & Laipple, 2020 ▸), was utilized. The illuminated volume is defined by the X-ray beam size and the thickness of the micropillar along the beam direction. The scattering patterns represent the combined signal from all scattering elements within the illuminated volume. In most cases (∼90%), a single dominant reflection with significantly higher intensity than the others is observed. Given that the X-ray beam size is significantly smaller than the average grain size, the diffraction signal in most cases likely originates from a single or a few grains. The frequent observation of a single strongest reflection suggests that, within the illuminated volume, one grain (or phase/orientation) predominantly contributes to the diffraction. However, due to the complex relationship between intensity, orientation and phase content, further analysis would be required to conclusively associate diffraction intensity with volumetric dominance. Because the grain size exceeds the beam size, each scan position probes only a few grains, providing a representative local diffraction signal. The *qz* position was obtained from azimuthal integration and Gaussian fitting of the dominant reflection, which approximates the center of mass of the 3D diffraction spot with an accuracy in *d* spacing better than 0.05%. In this work, this reflection was selected to extract key structural parameters, including the peak position, full width at half-maximum (FWHM) and orientation.

The azimuthal integration of the intensity profile and subsequent mathematical fitting are shown in Fig. 3 ▸(*C*). The fitting was performed using a model comprising four Gaussian components, allowing accurate determination of the most intense peak. This method provides a stable and reproducible way to identify and analyze the dominant reflection, making it suitable for automated structural characterization. For subsequent analysis, 1D integrations were performed in both azimuthal and radial directions, as indicated by the black arrows in Fig. 3 ▸(*A*). To ensure the entire scattering signal was accounted for in both directions, the integration zones were selected to be maximally wide, as highlighted by the dashed polygons in Figs. 3 ▸(*A*) and 3 ▸(*B*). This is a suitable approach because the employed X-ray detector is free of noise. This approach was applied to compare the scattering patterns at the same location on the micropillar before [Fig. 3 ▸(*A*)] and after [Fig. 3 ▸(*B*)] compression, capturing changes induced by deformation. In both patterns, a set of X-ray peaks distributed along a circular trajectory is clearly visible, along with additional peaks located above and below the rings. In the pristine state [Fig. 3 ▸(*A*)], the peaks exhibit an almost circular shape, with some barely distinguishable shape variations. In contrast, after deformation at the same location—3 µm below the micropillar’s top surface and 5 µm from the left-hand edge—the peaks become more elongated along the circular trajectory. As shown in the integrated curves in Fig. 3 ▸(*C*), these peaks also shift slightly towards smaller *q* values. These observations indicate that deformation leads to a microtextured structure accompanied by lattice expansion of (2.11 ± 0.01)%.

From the integrated curves, two reflections were selected for further analysis: the *q* = 27.22 nm^−1^ reflection, corresponding to γ-TiAl 111 for the pristine state, and *q* = 31.63 nm^−1^, corresponding to γ-TiAl 200 for the deformed (Schuster & Palm, 2006 ▸) micropillar. Additional peaks attributed to α2-TiAl are also observed, such as those at *q* = 25.1 nm^−1^ and *q* = 34.95 nm^−1^, though their intensities are significantly lower than those of the γ-TiAl reflections. In the pristine state, the γ-TiAl 111 reflection dominates and was collected during a point-by-point scan through the 2D area of the top section of the micropillar, as shown in Fig. 1 ▸(*B*). However, in the deformed state, the γ-TiAl 200 reflection becomes more intense and exhibits a slight shift towards smaller *q* values. In reciprocal space, larger *q* values correspond to smaller lattice spacings in real space. Thus, a shift to lower *q* values indicates lattice expansion, which is consistent with the observed deformation effects. These results provide critical insights into the effects of uniaxial compression on the polycrystalline TiAl micropillar, revealing structural changes such as microtexture and lattice distortion. The diffraction patterns represent discrete Bragg reflections from individual grains rather than full Debye–Scherrer rings, due to the coarse grain size and limited angular coverage. The strongest reflections were indexed as γ-TiAl 111 and 200, and their measurement location is marked in Fig. 1 ▸(*B*).

Utilizing the same analytical approach, the strongest reflections across all radial angles were identified through Gaussian fitting [Fig. 3 ▸(*D*)]. High-resolution data were collected throughout the entire scanned area, allowing for the estimation of the orientation distribution within the upper section of the micropillar.

**Figure 4:**
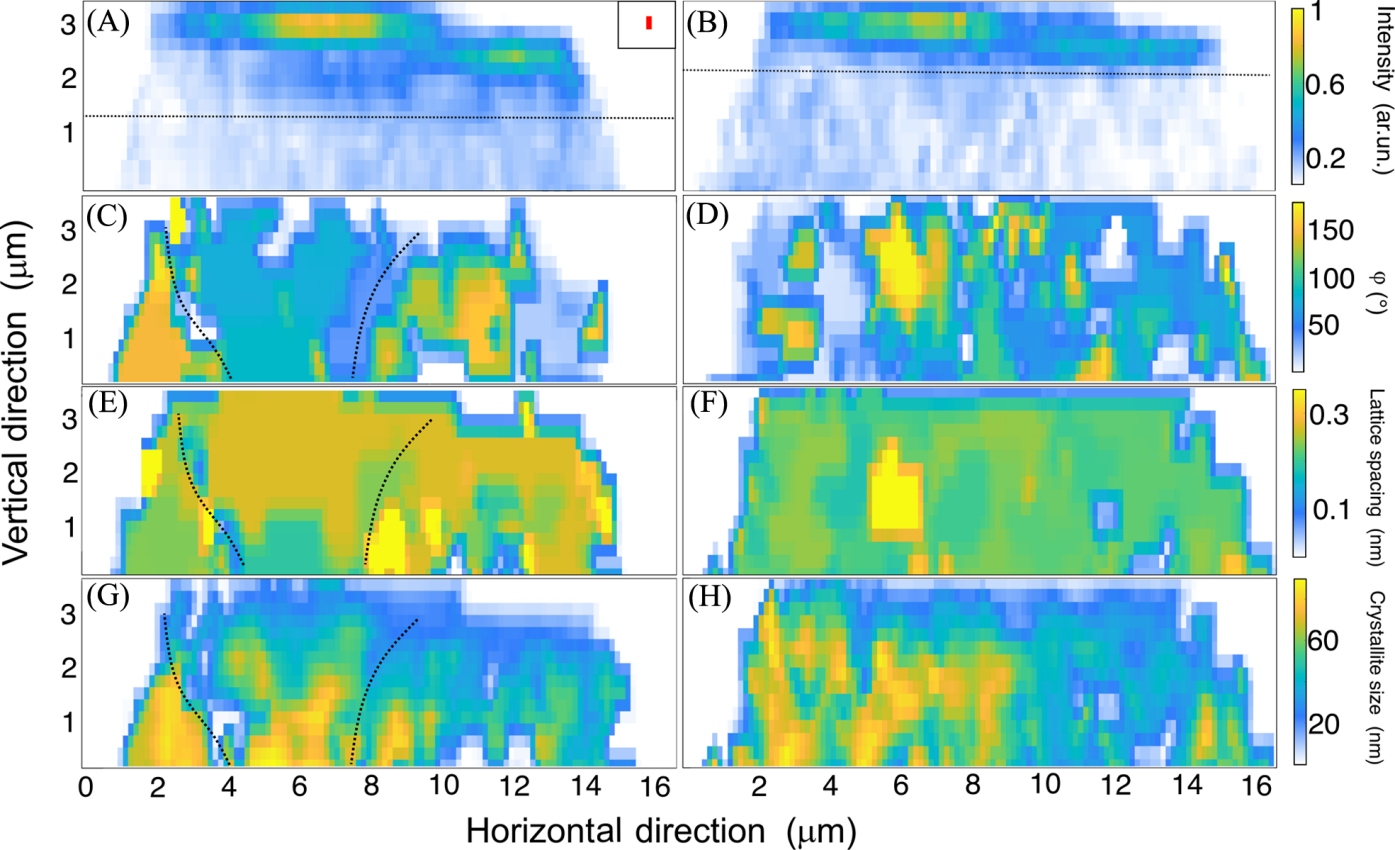
X-ray fluorescence map of Xe^+^ distribution in the top part of the micropillar (*A*) before and (*B*) after compression, 2D maps of the orientation spread (*C*) before and (*D*) after compression, the lattice spacing (*d*) distribution (*E*) before and (*F*) after compression, and the crystalline size (*G*) before and (*H*) after compression.

All the described data and characteristics were obtained from a single 2D raster scan. On the basis of these data, the structural properties of the micropillar can be characterized in terms of the local orientation distribution, size, lattice spacing, and the characteristic dimensions of structural components and their variation after compression. The correlation between X-ray fluorescence data, revealing the Xe^+^ ion distribution in the top part of the micropillar, and X-ray nanodiffraction data, analyzed using our new approach, is presented in Fig. 4 ▸. Obtained data were translated into real-space values from reciprocal ones following Pietsch *et al.* (2004 ▸). The figure illustrates both states of micropillar deformation: pristine and compressed.

Figs. 4 ▸(*A*) and 4 ▸(*B*) present two-dimensional maps of the Xe^+^ ion signal distribution in the upper region of the micropillar before and after compression, respectively. The Xe ion distribution maps demonstrate that the implanted ion ‘cloud’ largely retained its original shape after compression, with two distinct regions of higher concentration along the left-to-right axis. Compression resulted in a decrease in signal intensity, suggesting a slight redistribution of Xe^+^ ions. The observed redistribution of Xe^+^ ions after compression may be attributed to stress-assisted ion migration and defect formation, which promote ion mobility near grain boundaries and dislocation sites. Additionally, partial ion loss due to sputtering or relaxation effects under mechanical strain has been reported in FIB-milled metallic systems (Zhou *et al.*, 2025 ▸). Despite this redistribution, the highest concentration remained on the left side. Additionally, the depth of the Xe-enriched layer decreased from 1.8 µm before deformation to 1.2 µm post-compression. These observations suggest localized structural modifications due to compression.

Figs. 4 ▸(*C*) and 4 ▸(*D*) illustrate the grain orientation distribution in the same micropillar region before and after compression, respectively. In the pristine state, three distinct grains are discernible along the left-to-right axis (demarcated by dotted lines), each characterized by a specific crystallographic orientation. The left grain exhibits an orientation angle of 148°, the middle grain, which correlates well with the region of higher Xe content and contains minor inclusions, has an orientation of 76°, while the rightmost grain, spanning between 9 and 12 µm in the horizontal direction, displays an orientation angle of 140°. Post-compression, the grain structure exhibits considerable changes, with an increased number of smaller grains and multiple orientation shifts. These changes are particularly pronounced in the former middle grain, which transitions from a single grain structure in the pristine state to a more heterogeneous spotty multigrain configuration dominated by an orientation angle of 144°. Additionally, the Xe-enriched layer now correlates with a more granular and gradient-like orientation distribution, suggesting deformation-induced grain refinement.

Integration of nanodiffraction data in the radial direction provides quantitative insights into lattice periodicity, which can be correlated with the grain orientation and X-ray fluorescence data. Fig. 4 ▸(*E*) depicts the distribution of lattice spacing for the pristine micropillar. The map appears largely homogeneous, except for the leftmost region, corresponding to the first grain in the previous orientation map, where the lattice spacing is slightly reduced to 0.20 nm compared with the 0.22 nm observed in the other two grains. Additionally, inclusions with a *d*-spacing value of 0.27 nm are visible, likely corresponding to smaller separate grains embedded within the microstructure. After compression, the scanned area exhibits a more uniform distribution of *d* spacing, with a dominant value of 0.21 nm, except for a central region where the *d* spacing increases to 0.23 nm. The *d*-spacing maps represent the strongest Bragg reflection at each scan point, which may correspond to different *hkl* reflections depending on local grain orientation and phase. These maps are intended to highlight point-by-point structural changes rather than intra-grain variations. These findings, in conjunction with prior observations, support the conclusion that the micropillar transforms into a multigrain, but structurally more homogeneous, region post-deformation [Fig. 4 ▸(*F*)]. This numerical characterization is consistent with values obtained from transmission electron microscopy investigations of the same material in the pristine state, as reported by Fröbel & Laipple (2020 ▸). The FWHM of the dominant Bragg reflections was used to estimate local crystallite size, revealing structural disorder and grain refinement. Before compression [Fig. 4 ▸(*G*)], the crystallite-size distribution shows distinct regions with smaller domains (∼20–30 nm) and larger grains (∼50–60 nm). After compression [Fig. 4 ▸(*H*)], the distribution becomes more uniform, with a shift toward smaller average domain sizes, indicating increased defect density and fragmentation due to plastic deformation.

## 4. Discussion

Fröbel & Laipple’s (2020 ▸) findings regarding the mechanical properties of the investigated TiAl alloy configuration suggest that crucial structural changes can be expected after a 10% compression test. Changes in the scattering-intensity distribution are observed, although a phase transformation is not anticipated under the applied load and stable ambient conditions. Several factors may explain the change in dominant orientations. First, under external force, grains may reorient to align more favorably with the applied load, reducing the peak intensity of the pristine configuration and increasing the intensity of newly aligned orientations (Lim *et al.*, 2022 ▸). Second, plastic deformation, as shown by SEM studies revealing cracks on the micropillar surface and a height reduction from 28 to 26.4 µm (Fig. 1 ▸), can concentrate defects in planes of the dominant orientation, leading to decreased peak intensity (Cui *et al.*, 2021 ▸). Strain fields generated within the structure may also shift the peak position in *q* space [Fig. 3 ▸(*C*)]. Third, planes with the highest resolved shear stress are more likely to deform preferentially, and this slip may alter the crystal structure along these planes, diminishing the diffraction intensity of the original strongest peak if it corresponds to one of the active slip systems (Smallman & Bishop, 1999 ▸). In addition, a lattice expansion of ∼2% was detected. Although this value appears high for a small pillar expected to be relaxed by its free surfaces, the polycrystalline nature of the pillar must be considered. Strain incompatibilities at grain boundaries, defect accumulation and intergranular constraints can induce localized lattice distortions, while surface relaxation, minor compositional variations or measurement uncertainty may further contribute. Therefore, the observed lattice change is interpreted as a localized structural distortion rather than a homogeneous residual elastic deformation. Together, these mechanisms contribute to the observed intensity redistribution and peak shifts.

To better understand the internal structure of the deformed micropillar and to support interpretation of the SXND results, we performed an EBSD analysis on a single micropillar central cross-sectional slice, selected near the top of the micropillar. This region was expected to exhibit stronger effects of compression, making it a suitable position to assess local microstructural features such as grain size, defect density, orientation spread and phase distribution. EBSD was not used for direct comparison with SXND. Rather, it provides a qualitative structural reference to help contextualize the diffraction-based findings. The EBSD scan, covering only a 5 × 5 µm area [marked in red in Fig. S2(*A*)], was completed within a time frame comparable to that of the 2D SXND scan (∼3 h) shown in Fig. 1 ▸(*B*). However, the EBSD measurement represents only a single 2D slice and does not provide volumetric information. A more comprehensive EBSD-based structural analysis would require multiple slices across a larger area, while full 3D information from SXND would require tomographic reconstruction from multiple angular projections.

The fabricated micropillar exhibits a slight taper resulting from the Xe^+^ plasma FIB milling process, with the top diameter (∼10 µm) being smaller than the base diameter by ∼10–15%. Such tapering can influence stress distribution during compression, leading to a slightly higher apparent yield strength and reduced plastic strain compared with a pillar with parallel sidewalls (Uchic *et al.*, 2004 ▸). Nevertheless, the overall deformation behavior observed here (a high yield stress followed by limited strain hardening) remains consistent with previous reports for γ-TiAl micropillars of comparable dimensions and taper angles. Regarding the X-ray nanodiffraction measurements, the variation in cross-sectional area along the pillar height causes minor changes in the illuminated volume and local incidence geometry. However, because the probed region (upper ∼10 µm of the pillar) is small compared with the total height and the X-ray beam is submicrometre in size, the taper does not significantly affect the observed diffraction peak positions or the interpretation of local structural modifications.

The SXND analysis presented here is based on the characterization of the strongest Bragg reflection in the scattering pattern at each scan point. This allowed us to generate structural maps of grain domain size, orientation distribution and phase variation across the upper region of the micropillar, as shown in Fig. S1. Changes observed in these parameters along the pillar height reflect the structural evolution caused by mechanical compression. In the top part, where deformation is strongest, we observe smaller diffraction domains and broader orientation spread. Toward the base of the mapped region, larger and more uniform domains are apparent, with reduced orientation dispersion. These trends are consistent with the EBSD data from the nearby cross section, which show smaller, more fragmented, grains with higher defect density near the top and coarser grains in the lower part of the section. Additionally, SXND revealed variations in *d*-spacing values indicative of at least two phases, as discussed before [Fig. 3 ▸(*C*)].

Critically, the success of SXND in this work is largely due to the specific grain configuration within the micropillar. The average grain size, of the order of 3–5 µm, is significantly larger than the ∼300 nm X-ray beam, meaning that only two to three grains are illuminated at any given scan point within the ∼10 µm diameter of the pristine micropillar. This favorable grain-to-beam size ratio allows for the extraction of discrete and interpretable diffraction signals based on the strongest Bragg reflection, enabling mapping of microstructural variations despite the lower spatial resolution compared with EBSD. Thus, while not universally applicable, the presented approach for the SXND data analysis proves to be a productive tool for internal structure analysis in microscale specimens with sufficiently coarse grain structures. It provides rapid volumetric structural information without altering the sample, making it especially valuable for *in situ* or *operando* applications.

## 5. Conclusions

This study demonstrates a fast non-destructive approach to analyze structural changes in microscale specimens using SXND. Applied to a γ-TiAl-based micropillar fabricated via coarse Xe^+^ FIB milling and subjected to uniaxial compression, SXND effectively captured submicrometre-scale structural evolution and grain-level deformation. In cases where the X-ray beam size is significantly smaller than the grain size and the initial crystallographic orientation is known, the strongest Bragg reflection can be reliably used to represent the local illuminated volume. The method successfully quantified plastic deformation effects in terms of grain-size reduction, orientation spread and phase variation. Its capacity to provide rapid volumetric structural information without altering the sample makes it particularly suitable for *in situ* and *operando* studies of heterogeneous polycrystalline materials. These findings underscore the potential of peak-based SXND analysis as a robust and time-efficient tool for real-time investigation of complex microstructures, especially in cases where conventional techniques may fall short due to spatial or temporal constraints.

## Supplementary Material

Supporting information. DOI: 10.1107/S160057672500946X/gue5011sup1.pdf

## Data Availability

Data will be made available on request.
