# Supplementary material for: Efficient detection of deformation-induced microstructural modifications in polycrystalline micropillars using scanning X-ray nanodiffraction
Source: J Appl Crystallogr. 2026 Feb 1;59(Pt 1):85–92. doi: 10.1107/S160057672500946X (PMC12871479; doi:10.1107/S160057672500946X)
Supplement: Supplementary file 1 [file j-59-00085-sup1.pdf]

JOURNAL OF APPLIED CRYSTALLOGRAPHY

Volume 59 (2026)

Supporting information for article:

Efficient detection of deformation-induced microstructural modifications in polycrystalline micropillars using scanning X-ray nanodiffraction

Anton Davydok, Kritika Singh, Surya Snata Rout and Christina Krywka

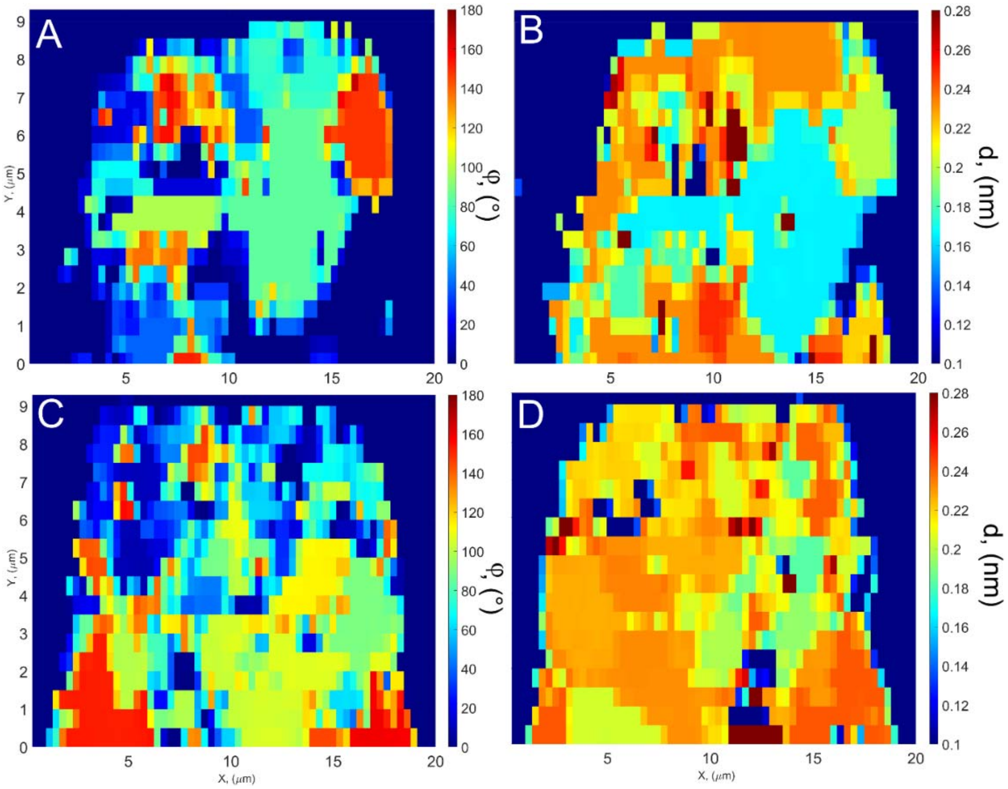

Figure S1 (A,B) complete maps of the orientation distribution and the lattice spacing (d) at pristine state; (C,D) – at the deformed state

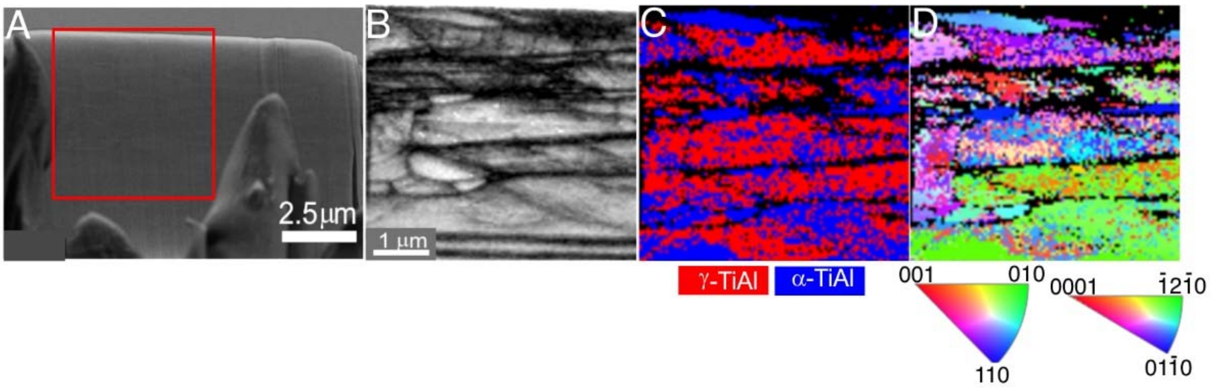

Figure S2 A) SEM image of the micropillar top part, with red rectangle is marked area where EBSD analysis was performed; results of EBSD analysis: B) band contrast image; C) phase distribution; D) orientation distribution
